# Functionality of Intergenic Transcription: An Evolutionary Comparison

**DOI:** 10.1371/journal.pgen.0020171

**Published:** 2006-10-13

**Authors:** Philipp Khaitovich, Janet Kelso, Henriette Franz, Johann Visagie, Thomas Giger, Sabrina Joerchel, Ekkehard Petzold, Richard E Green, Michael Lachmann, Svante Pääbo

**Affiliations:** 1 Max Planck Institute for Evolutionary Anthropology, Leipzig, Germany; 2 Institute for Computational Biology, Shanghai Institutes for Biological Sciences, Chinese Academy of Sciences, Shanghai, China; North Carolina State University, United States of America

## Abstract

Although a large proportion of human transcription occurs outside the boundaries of known genes, the functional significance of this transcription remains unknown. We have compared the expression patterns of known genes as well as intergenic transcripts within the ENCODE regions between humans and chimpanzees in brain, heart, testis, and lymphoblastoid cell lines. We find that intergenic transcripts show patterns of tissue-specific conservation of their expression, which are comparable to exonic transcripts of known genes. This suggests that intergenic transcripts are subject to functional constraints that restrict their rate of evolutionary change as well as putative positive selection to an extent comparable to that of classical protein-coding genes. In brain and testis, we find that part of this intergenic transcription is caused by widespread use of alternative promoters. Further, we find that about half of the expression differences between humans and chimpanzees are due to intergenic transcripts.

## Synopsis

In order to convert the genetic information encoded in an organism's genomic sequence into the functional features, the genomic sequence must be transcribed. According to the current genome annotation, the human genome encodes 20,000–25,000 protein-coding transcripts and a smaller number of non-coding transcripts. There is, however, a growing body of evidence indicating that a much greater proportion of the human genome is transcribed than is accounted for by the existing annotation. Much of this evidence has been found using tiling arrays, microarrays that enable the measurement of transcription regardless of existing annotation. Although some have suggested that these transcripts represent previously unidentified functional RNAs as well as extensions of known genes, the extent of their functionality remains unknown. In this study, Khaitovich et al. assess the functionality of these novel transcripts by testing the extent to which their expression is conserved between humans and chimpanzees in different tissues. The results suggest that, surprisingly, the expression of both known and novel transcripts was affected by the same functional constraints during human and chimpanzee evolution.

## Introduction

RNA transcription is the first step in transferring the information encoded in a genome sequence into the phenotypic features of an organism. Naturally, much effort has been spent determining which regions of the human genome are transcribed and for what biological purpose. Until recently, these efforts were largely restricted to either computational gene prediction or alignment of sequenced cDNAs and ESTs to the genomic sequence. Based on information collected using these methods, the human genome annotation is converging on a set of 20,000–25,000 protein-coding genes and a smaller set of non-coding RNAs. However, there is a growing body of evidence acquired using a variety of new approaches that indicates that much more transcription occurs than is accounted for by the existing annotation [1–3].

Tiling arrays are a powerful new tool for detecting transcription in an unbiased manner [2,4–6]. On such arrays, probes are placed at regular intervals across the entire genome or a region of interest, regardless of annotation or previous evidence of transcription. This design allows for discovering transcription without any prior expectation as to where it might occur. Several studies employing tiling arrays show that in humans, as well as in other organisms, a large amount of RNA transcription occurs outside of previously annotated genes [2,6–11]. While biological roles have yet to be determined for much of this transcription, some features of this intergenic transcription are becoming apparent.

In general, intergenic transcripts tend to be expressed at low levels, sometimes at or below the detection limit of Northern blot or RT-PCR techniques [2,9,12]. In many cases, but not always, these transcripts are located close to annotated genes or even within genes, in introns or on the opposite DNA strand [6,9,10,13]. This physical proximity suggests that many intergenic transcripts may simply be unannotated exons of known genes, a suggestion that has been confirmed for a subset of these transcripts [6,9,10,13]. Finally, and perhaps most surprisingly, most human intergenic transcripts are not conserved on the DNA sequence level when compared to mouse [6,9,12]. Furthermore, there is no evidence for expression conservation in intergenic regions that are conserved between human and mouse on the DNA sequence level [14].

This lack of sequence conservation has led to the suggestion that intergenic transcripts represent the products of stochastic and unproductive activity of the RNA transcription machinery and are consequently degraded [15]. However, a lack of conservation on the DNA sequence level does not preclude the possibility that these transcripts are conserved at the expression level. Yet, to date, the evolutionary conservation of intergenic expression has not been investigated. Previous work has shown that human and chimpanzee expression comparisons can be used to estimate the amount of selective constraints (negative selection) and positive (adaptive) selection occurring on transcripts of known genes in different tissues [16]. To determine the extent of positive selection and constraint on intergenic transcripts, we used tiling microarrays to assay the expression levels of known and intergenic transcripts in four human and chimpanzee tissues.

## Results

### Transcription across the ENCODE Regions

We have analyzed the evolutionary conservation of expression patterns between transcripts of known genes, as well as transcripts derived from intergenic regions in humans and chimpanzees, by measuring transcription in a strand-specific manner from both DNA strands in the 44 genomic regions studied by the ENCODE consortium covering approximately 1% of the human genome (to which we will refer as the ENCODE regions) in four tissues (brain, heart, testis, and lymphoblastoid cell lines) from five humans and five chimpanzees, using tiling arrays based on the human genome sequence (Table S1 and Materials and Methods). In order to exclude any hybridization differences between human and chimpanzee samples caused by nucleotide sequence differences between the species, we removed from the analysis all array probes that do not match the chimpanzee genome sequence with 100% identity over their entire length. Further, we excluded all array probes corresponding to annotated human pseudogenes in order to exclude possible cross-hybridization with transcripts derived from the parental functional genes (Materials and Methods).

We classified the remaining probes as exonic, intronic, or intergenic using the GENCODE annotations provided by the ENCODE consortium [17]. We defined exonic and intronic probes as those complementary to the transcribed strand of known genes (Materials and Methods). By contrast, we define intergenic probes as those mapping outside known genes without regard to strand, in order to avoid expression signals caused by labeling artifacts arising from spurious second-strand cDNA synthesis [18]. It should be noted that in doing so, we exclude antisense transcripts from genic regions from the analyses described.

**Figure 1:**
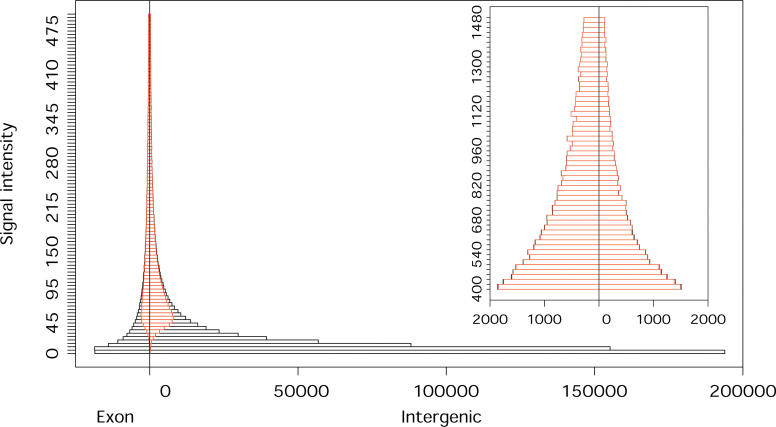
Signal Intensities of Exonic and Intergenic Probes The signal intensity range presented in the main figure covers 95% of all array probes with positive signal intensity. The insert shows the signal intensity range including the additional 4% of array probes. The *x*-axis shows the number of exonic and intergenic probes. Red indicates the proportion of probes we classified as expressed. Since there exists no empirically established cutoff for classifying tiling array probes into “expressed” and not expressed, we chose an arbitrary cut-off based on both absolute signal intensity and relative expression of perfect match and mismatch probes (Materials and Methods). The distribution presented here is based on an average of the four tissues. Taken separately, all tissues show very similar distributions (Figure S1).

In order to compare the expression of genic and intergenic regions, we did not follow the approach of grouping probes into transcriptional units according to their expression behavior [2,6,9]. Instead we conducted the entire analysis at the level of individual probes. Although this approach precludes us from identifying individual transcripts, it allows for comparison of genic and intergenic expression on the same basis, without bias toward a particular transcript length or signal intensity. We first compared the relative expression levels of known and intergenic transcripts by examining the distribution of signal intensities for exonic and intergenic probes (Figure 1). In agreement with previous studies [19], we find that the major proportion of expressed probes lies in intergenic regions, but that a large portion of this intergenic expression is of low signal intensity compared with that of known genes (Figure 1). However, not all intergenic probes have a low intensity. In fact, since the overall number of intergenic probes is greater than that of exonic probes, comparable numbers of exonic and intergenic probes are expressed at higher intensities. The only exception to this is the 5% of probes with the highest expression, where exonic probes predominate.

**Figure 2:**
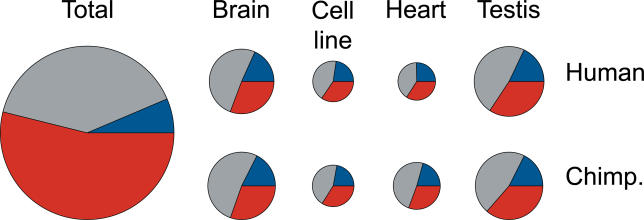
Distribution of Expressed Probes among Exons, Introns, and Intergenic Regions The distributions of expressed probes among exonic (blue), intronic (gray), and intergenic (red) regions in four tissues in humans and chimpanzees. The size of the circles reflects the number of probes (Table S2). All distributions represent an average of the two DNA strands, measured independently. There were no identifiable differences between expressed probe distributions on the two strands (Figure S2). “Total” indicates the distribution of all array probes, irrespective of their expression levels.

Further, although the total numbers of expressed probes differ among tissues, with brain and testis having on average 20% of probes classified as expressed, and heart and lymphoblastoid cell lines having on average 12% of probes classified as expressed, we find that the relative proportions of expressed exonic, intronic, and intergenic probes in humans or in chimpanzees are similar in all tissues (Figures 2 and S2, Table S2). Namely, in each tissue, approximately 20% of expressed probes fall in exons, 45% in introns, and 35% in intergenic regions (Table S2). Therefore, the largest proportion of expressed probes is located in introns. This is not surprising, given that we examined expression in total cellular RNA samples, which may have included a substantial proportion of partially processed or unprocessed transcripts (Materials and Methods). Since we cannot correct for these effects in our analysis, we limited the following analyses to probes located within exons and intergenic regions, respectively.

Compared with the total distribution of array probes (6% in exons, 40% in introns, and 54% in intergenic regions), a greater proportion of exonic probes and a lesser proportion of intergenic probes are classified as expressed. Still, in each tissue we find on average 2.4 times more expressed intergenic than expressed exonic probes (Table S2). It should be noted, however, that the ratio of expressed exonic and intergenic probes depends a great deal on the definition of expression, i.e., on the cut-off chosen (Figure 1).

**Figure 3:**
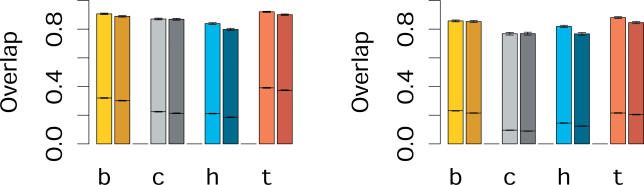
Overlap of Expressed Probes between Species Shown is the overlap of expressed exonic (left, lighter shades) and intergenic (right, darker shades) probes between humans and chimpanzees in brain (B), lymphoblastoid cell line (C), heart (H) and testis (T) for the positive (left panel) and negative (right panel) chromosome strands. The horizontal lines inside the bars show the overlap expected by chance. The error bars represent 95% confidence intervals based on bootstrapping of 1,000 subsets of exonic and intergenic probes having the same number of probes from each category and the same signal intensity distribution.

We next investigated whether the same probes are expressed in humans and chimpanzees in each tissue. This is important, because if intergenic expression is stochastic in nature, we might expect to find similar amounts of transcription but little overlap between the intergenic probes expressed in two species. Since the reliability of expression detection depends greatly on signal intensity, and exonic and intergenic probes have different signal intensity distributions, we limited our analysis to exonic and intergenic probes chosen to have the same distributions of signal intensity (Figure S3 and Materials and Methods). We find that, among this set of expressed intergenic probes, 76%–90% overlap between humans and chimpanzees. This is 3- to 8-fold more overlap than what would be expected by chance. These high degrees of overlap are seen in all four tissues (Figure 3). By comparison, 77%–92% of expressed exonic probes overlap between the species. Furthermore, we find a similar degree of overlap between expressed intergenic and expressed exonic probes when comparing different tissues in humans and chimpanzees (Figure S4). Thus, sites of intergenic expression are not randomly distributed in the genome, but show almost as much conservation with respect to their location as that of exonic expression. However, this does not necessarily indicate that intergenic expression is of functional significance, since it is entirely conceivable, for example, that certain intergenic sequences will by chance have a high affinity to the RNA transcription machinery and thus trigger spurious transcription. Because of the high degree of DNA sequence similarity between humans and chimpanzees, such spurious transcription would be largely shared between humans and chimpanzees.

### Transcriptome Evolution

**Figure 4:**
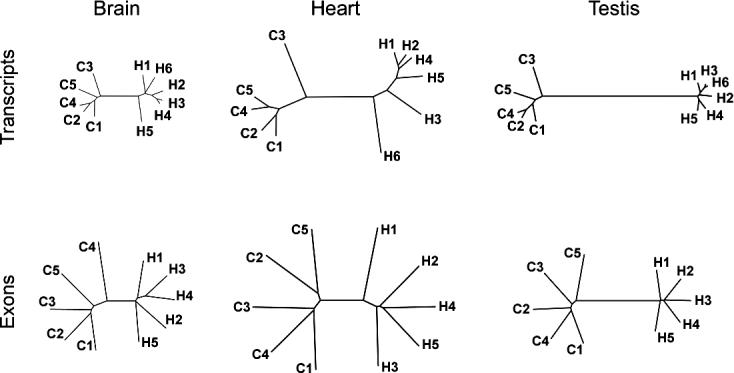
Schematic Representation of Expression Diversity and Divergence in Humans and Chimpanzees in the Three Tissues The expression was measured using either “classical” transcript-based arrays (upper row) or exonic probes on the tiling arrays (lower row). The trees are inferred from the mean of the squared difference of expression intensities of all detected probe sets [16] or all expressed exonic probes (Materials and Methods). Greater variation observed within species for the exonic probe expression is likely due to greater technical variation associated with tiling arrays measurements, caused by probe design limitations, and by the fact that tiling array measurements are based on signal probe intensity and not on the cumulative intensity of a set of probes.

Previous studies have indicated that genes expressed in different tissues experience different extents of negative and positive selection on both DNA sequence and gene expression levels [16,20]. On the gene expression level, this is reflected in differences in diversity and divergence patterns among tissues. Among the tissues studied to date, brain and testis differ the most. That is, in brain, negative selection acts most strongly, leading to significantly reduced expression divergence between species. By contrast, in testis the expression divergence-to-diversity ratio is significantly greater than in the other tissues, probably due to positive selection acting on the male reproductive system [16]. We first tested whether these observations hold true for the exonic transcripts measured on the tiling arrays. We find the same qualitative differences in the diversity and divergence patterns for the expression of exonic probes among the tissues used in both studies, i.e., brain, heart, and testis (Figure 4). Thus, tiling arrays can detect the evolutionary differences in tissue expression patterns seen previously using arrays designed to detect protein-coding transcripts.

**Figure 5:**
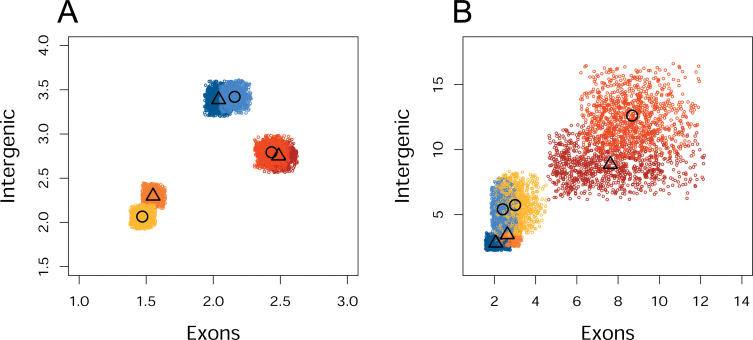
Expression Divergence and Divergence-to-Diversity Ratio in the Three Tissues for Exonic and Intergenic Probes Shown are the average expression divergence (A) and divergence-to-diversity ratio (B) between humans and chimpanzees in brain (yellow), heart (blue), and testis (red) for exonic and intergenic probes. The colored areas indicate 95% confidence intervals based on bootstrapping 1,000 subsets of exonic and intergenic probes, having the same number of probes from each category and the same signal intensity distribution. The darker shades indicate expression from the positive DNA strand, while the lighter shades indicate expression from the negative DNA strand. The symbols represent the mean value for each tissue on either the positive (▵) or the negative (○) strand, respectively.

We then investigated whether differences in diversity and divergence can also be observed for intergenic transcripts. Since we assume that these differences in transcript expression observed among tissues arise due to differences in the extent of functional constraints and positive selection in these tissues, in the absence of any function, no such differences would be expected. Thus, if we assume as a null hypothesis that intergenic transcripts have no function and represent products of spurious activity of the RNA transcription machinery, we expect that their expression diversity and divergence patterns should be the same among the three tissues. We therefore tested whether this is the case by comparing intergenic and exonic probe expression patterns in the three tissues. In order to avoid artifacts caused by differences in technical measurement errors associated with different probe signal intensities, we compared exonic and intergenic probes having the same signal intensity distribution (Figure S3 and Materials and Methods). In all three organs, we observe a 1.1- to 1.7-fold greater expression divergence between humans and chimpanzees for intergenic than for exonic probes, indicating that intergenic transcripts tend to evolve faster between species than exonic transcripts (Table S3). However, when we compare the diversity and divergence patterns based on the intergenic probe expression among the three tissues, we find that the brain shows less divergence than the other two organs (Figure 5A), while testis shows a greater divergence-to-diversity ratio (Figure 5B) to an extent comparable with the results for exonic probes. This finding contradicts our null hypothesis and suggests that intergenic transcripts are subject to functional constraints and positive selection at the same relative extents in these tissues as are exonic transcripts. This, in turn, indicates that much of intergenic transcription performs as-yet uncharacterized functions [19].

### Characteristics of Intergenic Transcription

A potential alternative explanation for the observed difference in intergenic expression patterns among tissues is cross-hybridization of transcripts of known genes to intergenic probes. We tested whether this may be the case by consecutively removing all probes that map to more than one location in the human genome with zero, one, two, three, four, or five mismatches, respectively. Although this procedure does not necessarily remove all probes that could cross-hybridize to transcripts of known genes, we would expect the difference between tissues seen in Figure 5 to become substantially and progressively reduced if cross-hybridization were the main cause of this effect. However, we find no such reduction (Table S3), either for divergence or for the divergence-to-diversity ratio. Further, if the bulk of observed intergenic transcription was the result of cross-hybridization, we would expect the expressed intergenic probes to be distributed randomly within intergenic regions. However, we find that although exonic probes show substantially greater clustering than intergenic probes, probably due to their higher average signal intensities, in agreement with previous studies [6,9,14], the expressed intergenic probes cluster significantly in all four tissues (Figure S5).

**Figure 6:**
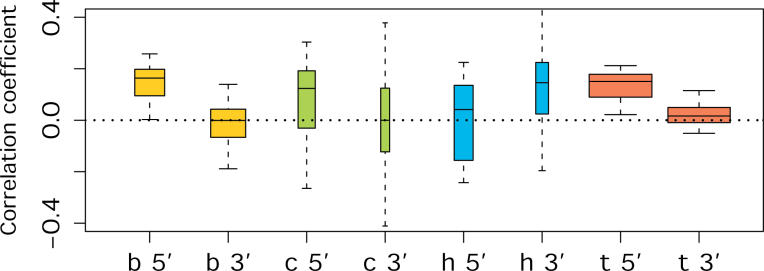
Correlation between Signal Intensity Difference of Intergenic Probes and that of the Nearest Exon Letters and colors indicate tissues (B, yellow—brain; C, green—cell line; H, blue—heart; T, red—testis). Correlation was calculated separately for probes located upstream (5′) or downstream (3′) from the nearest exon. The width of the bars is proportional to the number of the ENCODE regions showing significant correlations (Spearman correlation test, *p* < 0.05, corrected for multiple testing). The mean of the bars shows the mean correlation coefficient, while the bar borders represent a 75% confidence interval. The error bars depict a 95% confidence interval of the correlation coefficient, calculated by bootstrapping the list of intergenic probes within each region 500 times (Materials and Methods).

It has been previously reported [6] that intergenic transcripts tend to be located close to known genes. In agreement with this, we see significantly shorter distances than expected by chance (Wilcoxon test, *p* < 10^−6^) between expressed intergenic probes and the nearest exon in all four tissues. This may indicate that some intergenic expression arises from as-yet unannotated extensions of known genes. In order to test if this could be the case, we calculated the correlation between the signal intensity of intergenic probes with the signal intensity of the nearest exon. Overall, we find no such correlation (Figure S6), whereas we find a significant positive correlation between the signal intensities of exonic probes and probes in neighboring exons. We then repeated the same analysis using signal intensity differences between humans and chimpanzees instead of absolute signal intensities. This partially alleviates the problem that probe intensities depend to a great extent on probe sequence and do not very accurately reflect actual expression levels of transcripts. Indeed, in this case, we find a positive correlation between intergenic probes and probes in the nearest exon in testis, and to a lesser extent, also in brain (Spearman correlation test, bootstrap *p* < 0.05), but not in heart (*p* = 0.39) nor in lymphoblastoid cell lines (*p* = 0.36). In both brain and testis, this positive correlation is seen for probes located upstream, but not downstream, of the nearest exon (Figure 6). A portion of the observed intergenic expression, at least in brain and testis, is therefore likely to be due to unannotated 5′ extensions of known genes. This suggests that genes expressed in brain and testis use alternative promoters more often than the other tissues studied, an observation supported by results from full-length cDNA sequence analysis showing that brain and testis have the largest numbers of tissue-specific putative alternative promoters [21]. The absence of a correlation at the 3′ end of the genes indicates that transcriptional “read through” from the known genes is not a major source of intergenic transcription, and that alternative polyadenylation is either less common than alternative promoter usage or is better annotated. The latter may be expected given the 3′ bias of most EST libraries.

**Figure 7:**
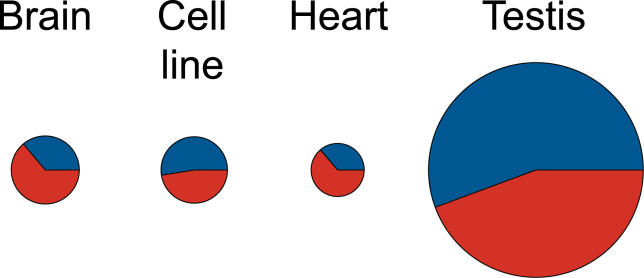
Proportions of Exonic and Intergenic Probes with Significant Expression Difference between Humans and Chimpanzees The colors indicate the proportions of exonic (blue) and intergenic (red) probes. The figure represents an average of the probe numbers identified as differently expressed on the positive and on the negative strands. The size of the circles reflects the number of probes.

Finally, we identified exonic and intergenic probes with significant expression differences between humans and chimpanzees (Student's *t*-test, *p* < 0.001, false discovery rate [FDR] < 5%). We find that in each individual tissue, about half of the total number of differently expressed probes originate in intergenic regions (Figure 7; Tables S4 and S5). In testis, both known and intergenic transcripts show three to four times more expression differences between the species than the other three tissues. This observation is consistent with previous studies [16], and reflects the large divergence-to-diversity ratio observed in this tissue for both exonic and intergenic probes.

## Discussion

Taken together, our results indicate that the expression of intergenic transcripts evolves under similar levels of functional constraint and positive selection as exonic transcripts. This observation implies that intergenic transcripts play functional roles, since no differences in the extent of positive and negative selection between tissues would be expected if the bulk of intergenic transcripts represented transcriptional “noise.” Any alternative explanation for the functionality of intergenic transcription would need to involve a mechanism that allows for a difference in a selective pressure on non-functional transcription in different tissues. Although such a scenario is not completely impossible, it is certainly not straightforward.

Although our results strongly suggest that intergenic transcripts have functional roles, numerous important questions remain. Firstly, in agreement with previous studies [6,9,12], we find no detectable differences in DNA sequence conservation between intergenic probes expressed in all four tissues, expressed in at least one tissue, and those not expressed at detectable levels (Figure S8 and Materials and Methods). In contrast, we find far greater DNA sequence conservation for exonic probes than for any of the intergenic probe categories (Figure S8). It should be noted, though, that these estimates of sequence conservation involve comparisons to distantly related species such as chicken, zebrafish, and fugu. Thus, it remains possible that sequence conservation may be discovered over a shorter evolutionary timespan. Future analysis involving comparisons to multiple primate species will be needed to address this question.

Secondly, it is not possible to quantitatively compare the extent of intergenic transcript functionality to that of exons of known genes. In all tissues we see similar, but somewhat greater, expression divergence of intergenic transcripts as compared to that of exonic transcripts (Figure 5, Table S3). Similarly, we see comparable, but somewhat smaller, differences between tissue expression patterns for intergenic transcripts than for exonic transcripts (Figure 5, Table S3). However, it is currently not clear if this is because a fraction of intergenic transcripts is not constrained at all, or if most intergenic transcripts are constrained but to a slightly lesser degree than exonic transcripts.

Thirdly, we note that most of our analysis considers only exonic and intergenic probes having the same signal intensity distribution. This analysis thus ignores a far greater proportion of intergenic than exonic transcription with low signal intensities. Although differences in evolutionary patterns between tissues can be observed for a large range of signal intensities for both intergenic and exonic probes (Figure S9), when we limit our analysis to low-signal-intensity probes, no obvious differences in evolutionary patterns between tissues is observed (Figure S10). Since technical noise increases rapidly with decreasing signal intensity, it is impossible to say whether this observation reflects a technical limitation of microarray technology or a biological phenomenon. An analysis of the possible functionality of low-intensity intergenic transcription must, therefore, await technical approaches allowing for more precise, high-throughput measurements of transcripts of with low signal intensities.

Finally, the fact that a proportion of intergenic transcription, at least in brain and testis, stems from 5′ extensions of known genes suggests that some intergenic transcripts evolve under similar selection pressures as known genes simply because they represent as-yet undiscovered parts of known genes. Since we find 5′ extensions of known genes mainly in brain and testis, they are unlikely to explain the bulk of intergenic transcription observed in all four tissues studied. Still, further work is necessary to clarify what proportion of intergenic transcription belongs to this category.

In summary, we show that intergenic transcripts are similar to exonic transcripts in the extent of functional constraints that they underlie in terms of their expression. Furthermore, we find that in each individual tissue, about half of the total number of probes differently expressed between humans and chimpanzees originate in intergenic regions (Figure 7). This suggests that intergenic transcripts might contribute to functional differences between the species to an extent comparable to exonic transcripts.

## Materials and Methods

### Tissue samples and microarray data collection.

Brain, heart, and testis postmortem samples were obtained from individuals who suffered sudden deaths for reasons other than their participation in this study and without any relation to the tissues used. All brain samples used in this study were dissected from the same area of the dorsolateral prefrontal cortex (Brodmann area 9) by the same person (PK). Human and chimpanzee samples were matched with respect to sex and relative age (Table S1). Total RNA was isolated from 100 mg of frozen tissue or 40 ml of liquid culture containing approximately 2.5 × 10^7^ of living cells using the TRIZol (Invitrogen, Carlsbad, California, United States) reagent according to the manufacturer's instructions and purified with MiniElute (Qiagen, Valencia, California, United States) kit following the manufacturer's instructions with no modification. All RNA samples used in this study were of high and comparable quality as gauged by the ratio of 28S to 18S ribosomal RNAs estimated using the Agilent 2100 Bioanalyzer system (Foster City, California, United States) (Table S1). The total RNA samples were subjected to a stringent DNAse treatment (incubation with 4 units of DNAseI per 20 μg of RNA at 37 °C for 30 min) to remove any possible traces of genomic DNA.

All samples were processed, labeled, and hybridized to Affymetrix (Santa Clara, California, United States) ENCODE 0.1 FORWARD and REVERSE arrays following the Whole Transcript Sense Target Labeling Assay protocol (http://www.affymetrix.com/support/technical/manuals.affx), with few modifications. Namely: 2 μg of total RNA were used in rRNA reduction protocol instead of the recommended amount of 1μg; 2.5 times greater than the recommended total reaction volume was used in the cDNA synthesis step; cDNA cleanup was carried out following standard phenol-chloroform extraction protocol; the entire amount of purified cDNA was used for the cRNA synthesis step carried out with Ambion Megascript kit (Austin, Texas, United States) according to the manufacturer's instructions for 16 h at 37 °C; and all RNA cleanup steps in the protocol were carried out with Qiagen MiniElute kit following the manufacturer's instructions with no modifications.

Samples for the FORWARD and REVERSE version of arrays were processed, labeled, and hybridized independently.

### Microarray data analysis.

Affymetrix microarray image data were acquired using an Affymetrix 3000 scanner with the default settings. The intensity of individual probes was calculated using standard Affymetrix GeneChip Operating Software. Resulting raw probe intensity files were analyzed using R (http://www.r-project.org). Prior to analysis we masked all oligonucleotide probes that are not 100% identical over their full length to both human (NCBI build 35) and chimpanzee (panTro1) genome sequences. This reduced the number of probes available for analysis from 755,455 to 503,459. We defined each probe as a pair of sequences, one perfectly matching the designated genomic sequence (PM) and another, with a transversion in the central position (MM).

All arrays used in this study were normalized together using “mas” background correction and scaling normalization procedures based on all remaining 503,459 probes. Expression values for each probe were calculated using the “affy” Bioconductor software package (http://www.bioconductor.org) as a difference between perfect PM and mismatch MM signal intensities.

We extracted gene annotations produced by the GENCODE working group (http://genome.imim.es/gencode) of the ENCODE consortium [17] for known genes, putative genes, and pseudogenes from the UCSC Genome Browser database (http://hgdownload.cse.ucsc.edu/goldenPath/hg17/encode/database/). Using these annotations, we assigned array probes to genomic regions corresponding to known genes, putative genes, and pseudogenes. We omitted probes falling within pseudogenes from further analyses. This further reduced the number of probes from 503,459 present on each strand to 498,556 on the positive and 498,527 on the negative strand. For each DNA strand, the remaining probes were separated into those mapping within exons and introns of known genes located on the relevant strand and intergenic probes. Intergenic probes were defined as mapping outside of the exons and introns of known genes on both strands. This excluded from analysis all potential antisense transcripts corresponding to genomic regions of known genes. However, the labeling protocol we used is known to produce spurious second-strand transcripts at the first cDNA synthesis stage, which would then be classified as “antisense transcripts” [18]. Our definition of intergenic region therefore excludes all such “shadow” transcripts of known genes that may be experimental artifacts. The numbers of probes on the positive and negative strands, respectively, were 30,994 and 32,216 for exonic; 202,205 and 202,821 for intronic; and 187,966 for intergenic regions. Parts of regions we classified as intergenic were annotated by the GENCODE working group (http://genome.imim.es/gencode) of the ENCODE consortium based on gene predictions as putative gene regions. However, excluding probes mapped within these regions did not affect any of our results (see, for example, Table S3 and Figure S5).

Expressed probes were defined as those with a PM signal intensity greater than the MM signal intensity in all five individuals of the same species, and with an average difference between PM and MM signal intensities in these five individuals greater than 50. The numbers of probes classified as expressed are shown in Table S2.

The overlap of expressed probes in exons and intergenic regions was determined as follows: First, we subsampled with replacement 1,000 times from the total of exonic and intergenic probes an equal number of probes having the same signal intensity distribution. Second, for each of the 1,000-probe subsets, we determined the proportion of expressed probes that overlap between humans and chimpanzees for exonic and intergenic probes independently. The overlap expected by chance was calculated using the overlap probability calculated using the same numbers of randomly chosen expressed and non-expressed probes in the two species.

Expression divergence between humans and chimpanzees was calculated as the mean squared difference between the mean human and the mean chimpanzee probe signal intensities. Expression variation was calculated as the mean probe signal intensity variation among five individuals of the same species. For each tissue, the trees representing expression distances were built using PHYLIP [22] based on squared mean expression differences for all probes expressed in a tissue calculated in all pairwise comparisons. In each tissue we observed clustering of all samples according to species for all classes of probes, such as exonic, intergenic, or intronic ones. The mean expression divergence and divergence-to-diversity ratio for each tissue, as well as the 95% confidence intervals, were calculated by subsampling with replacement 1,000 times from the total set of expressed exonic and expressed intergenic probes an equal number of probes having the same signal intensity distribution (an example is presented in Figure S3), and then calculating the expression divergence and divergence-to-diversity ratio for each subset. The same analysis was repeated after excluding all probes that map within putative genic regions as defined by the GENCODE annotation, or after excluding potential cross-hybridizing probes.

The clustering of probes was tested by calculating the genomic distances (based on probe positions according to NCBI build 35) between the two nearest probes for all intergenic and exonic expressed probes. The distribution of distances expected by chance was calculated by 1,000 permutations of expressed probe assignments within exonic and intergenic regions (Figure S5A). The same analysis was repeated after excluding all probes that map within putative genic regions (Figure S5B).

The proximity between expressed intergenic probes and known exons was tested within each of the 44 ENCODE regions by calculating the genomic distance between all expressed intergenic probes and the nearest annotated exon. Chance distribution was calculated by 1,000 permutations of expressed probe assignments within intergenic regions. The difference between the observed distance distributions for all regions was compared to that expected by chance using the Wilcoxon rank test.

The correlation between expressed intergenic probes and the nearest expressed exon, based either on the absolute signal or on the signal intensity difference between humans and chimpanzees, was calculated as follows: First, we calculated the mean signal intensity or mean signal intensity difference for each GENCODE known or known + putative exon, using all probes located within the exon. For each tissue, we defined the expressed exons as those containing at least one expressed probe. Further, for each expressed intergenic probe within each of the 44 ENCODE regions, we determined the nearest expressed exon by either disregarding their relative positions, or by separately determining the nearest exons located 5′ or 3′ from the intergenic probe. We then determined the Spearman rank correlation between the absolute signals or between the signal intensity differences of all expressed intergenic probes and probes in the nearest exon within each of the 44 ENCODE regions. Regions containing less than 100 expressed exonic probes were excluded from the correlation analysis. For each tissue, the mean correlation coefficient was based on the correlation coefficients for all regions showing significant correlation (*p* < 0.05, corrected for the number of regions used in analysis). For the results presented in Figure 6, in each tissue an average of 24 regions had 100 or more expressed exonic probes. Out of these regions, six and nine regions showed significant correlation in brain, three and one in cell lines, three and two in heart, and 16 and 16 in testis, for probes located upstream (5′) or downstream (3′) from the nearest exon, respectively. Repeating analysis using values from all ENCODE regions containing more than 100 expressed exonic probes did not change the results. The 95% confidence interval of the mean correlation coefficient was calculated by bootstrapping the expressed intergenic probes within each of the 44 ENCODE regions 500 times. The correlation analysis was separately applied to the signal intensity measurements of each DNA strand. The results obtained for the two strands were consistent throughout the analysis. The same approach was used in the correlation analysis of exonic probes and their neighboring exons.

Probes showing a significant expression difference between humans and chimpanzees were identified by applying the Student's *t*-test to the base two logarithm transformed signal intensities of human and chimpanzee samples of all probes expressed in a given tissue. An FDR corresponding to a given nominal significance cut-off was calculated by 1,000 random permutations of sample labels. For the chosen nominal significance cut-off (*p* < 0.001), FDR in all tissues was less than 5%. Genomic coordinates of the differently expressed probes on the human genome (Build 35) are listed in Table S5. We tested seven exonic and intergenic regions showing significant expression difference between humans and chimpanzees in brain and confirmed the array expression differences in six of these regions using quantitative PCRs (Figure S7).

### Masking probes with sequence differences between species and removing probes with the potential for cross-hybridization.

All probes on the ENCODE arrays were designed using the human genome sequence as a reference. To eliminate the effects of sequence divergence between humans and chimpanzees on the estimation of expression levels, we excluded from all analyses the array probes that do not match both the human (NCBI build 35) and the chimpanzee (panTro1) genome sequences with 100% identity over the entire probe length. Using BLAT [23], we aligned all Affymetrix probe sequences (http://www.affymetrix.com/support/downloads/library_files) to both the human and the chimp genomes. Oligonucleotide probes that matched both genomes perfectly were retained for the analysis while the probes with mismatches to either genome were masked.

Additionally, to study the potential effects of cross-hybridization, we removed from the set of perfectly matching probes those probes that could be aligned to more than one genomic location with zero, one, two, three, four, or five mismatches.

### DNA sequence conservation analysis.

DNA sequence conservation scores were based on the sequence conservation measures for each nucleotide position provided by the PhastCons conservation scores for eight-way multiple alignments between the human genome and genomes of the seven vertebrate species assemblies to the human genome build hg17, May 2004 (http://genome.ucsc.edu/goldenPath/help/phastCons.html). The seven species included in these alignments were: chimpanzee, dog, mouse, rat, chicken, zebrafish, and fugu. PhastCons uses maximum likelihood to fit a phylogenetic hidden Markov model to the sequence data of the different species. Each genome base is assigned a score reflecting the probability that the base position is in its most conserved state, according to the hidden Markov model. For each probe, the conservation score was calculated as a mean of all available PhastCons conservation scores for the bases within the probe. Probes with no available PhastCons conservation scores were removed from the analysis.

### Quantitative PCR.

Primer pairs were designed to match both the human and the chimpanzee genome sequence perfectly. PCR product length was chosen to lie between 150 and 300 bp. As a PCR template, we used purified cDNA prepared from total RNA extracted from the brain samples of three human and three chimpanzee individuals using either random hexamers or poly(dT) primer for the first strand cDNA synthesis. To control for possible amplification from genomic DNA, we used as a negative control cDNA prepared without the addition of reverse transcriptase during synthesis. As a positive control, we used PCR primers for a GAPDH transcript. The primers for this transcript were placed in different exons resulting in a PCR product of 602 bp in length if genomic DNA was amplified and a PCR product of 285 bp in length if cDNA was amplified. PCR products were visualized on agarose gels using ethidium bromide staining. The signal intensity of the PCR bands was quantified using ImageQuant (GE Healthcare, Chalfont St. Giles, United Kingdom). The signal intensity from the negative control lane was subtracted from the products' signal intensities to correct for unspecific amplification and signal background. Further, we normalized the products' signal intensities using the signal intensity of GAPDH transcripts measured four times in the six individuals used in Q-PCRs in order to correct for differences in the total amount of cDNA derived from each individual. The primers' sequence, their calculated melting temperatures, their corresponding positions in the human genome, the product size, the melting temperature, the GC content, and the expected product length are shown in Table S6.

## Supporting Information

Figure S1 Signal Intensities of Exonic and Intergenic Probes in the Four Tissues The distributions of signal intensities of exonic (left) and intergenic (right) probes are shown for brain (A), lymphoblastoid cell line (B), heart (C), and testis (D). The signal intensity range presented in each panel covers 95% of all array probes with positive signal intensity. The *x*-axis shows the number of exonic and intergenic probes. Red indicates the proportion of probes we classified as expressed.
(260 KB PDF)

Figure S2 Distribution of Expressed Probes from the Two DNA Strands among Exons, Introns, and Intergenic Regions The distribution of expressed probes among exonic (blue), intronic (gray), and intergenic (red) regions is shown in four tissues in humans (upper row) and chimpanzees (lower row) for the probes corresponding to the positive (A) and the negative (B) DNA strand. The “Total” indicates the distribution of all array probes irrespective of their expression levels. The size of the circles reflects the number of probes.
(391 KB PDF)

Figure S3 An Example of Subsampling of Equal Numbers of Expressed Exonic and Intergenic Probes Based on the Same Signal Intensity Distribution Shown are the original signal intensity distributions of exonic and intergenic probes expressed in human testis (A), and the signal intensity distributions of the probe subsets (B). The subsets of probes obtained using this procedure included on average 90% of exonic and 46% of intergenic probes from the original distributions.
(87 KB PDF)

Figure S4 Overlap of Expressed Probes between the Four Tissues The overlap between tissues is shown for expressed exonic (left) and intergenic (right) probes. The bars represent the proportion of overlap between expressed probes calculated in all pairwise comparisons among the four tissues for probes located on the positive (A) and on the negative (B) chromosome strands. The red colored areas inside the bars show the overlap expected by chance.
(149 KB PDF)

Figure S5 Distances between the Nearest Expressed Exonic and Intergenic Probes in the Four Tissues The distances were calculated either including (A) or excluding (B) probes mapped to putative genes. The distance distributions are shown separately for brain (upper left panel), lymphoblastoid cell lines (lower left panel), heart (upper right panel), and testis (lower right panel). For all tissues the distance range presented on the figure includes more than 90% of all distances. Red denotes the distance distribution expected by chance.
(486 KB PDF)

Figure S6 Correlation between Absolute Signal Intensity of Intergenic Probesor Exonic Probes and that of the Nearest Exon Correlations are shown for intergenic (left four bars) or exonic probes (right four bars). The letters and colors indicate tissues (B, yellow—brain; C, green—cell line; H, blue—heart; T, red—testis). The width of the bars is proportional to the number of the ENCODE regions showing significant correlations (Spearman correlation test, *p* < 0.05, corrected for multiple testing). The mean of the bars shows the mean correlation coefficient, while the bar borders represent a 75% confidence interval. The error bars depict a 95% confidence interval of the correlation coefficient calculated by bootstrapping the list of intergenic probes within each region 500 times.
(68 KB PDF)

Figure S7 Comparison of Expression Differences between Species Measured on Tiling Arrays and by Q-PCR Shown are expression differences between species in seven genomic regions measured on tiling arrays (red) and by Q-PCR using as a template cDNA generated using random primers (dark blue) or poly(dT) primer (light blue). Tested regions one and seven correspond to intergenic transcripts; the remainder, to the transcripts of known genes. The error bars represent the standard error of the mean.
(112 KB PDF)

Figure S8 Average DNA Sequence Conservation Scores for Intergenic and for Exonic Probes Shown are the average DNA sequence conservation scores (Materials and Methods) calculated for intergenic probes expressed in all four tissues (A), in at least one tissue (B), not expressed in any of the four tissues (C), and for exonic probes (D). The error bars depict a 95% confidence interval of the mean conservation score, calculated by bootstrapping the sequence conservation scores within each list 1,000 times.
(74 KB PDF)

Figure S9 Changes in Expression Divergence and Divergence-to-Diversity Ratio in the Three Tissues Depending on the Signal Intensity Shown are the average expression divergence (A and B) and divergence-to-diversity ratio (C and D) between humans and chimpanzees in brain (blue), heart (black), and testis (red) for exonic (A and C) and intergenic (B and D) probes measured in a sliding window within a signal intensity range from 50 to 1200.
(323 KB PDF)

Figure S10 Expression Divergence and Divergence-to-Diversity Ratio in the Three Tissues for Low-Intensity Signals Shown are average expression divergence (A) and divergence-to-diversity ratio (B) between humans and chimpanzees in brain (yellow), heart (blue), and testis (red) for exonic and intergenic probes with a signal intensity lower than 50 in both species. The colored areas indicate 95% confidence intervals based on bootstrapping 1,000 subsets of exonic and intergenic probes. The darker shades indicate expression on the positive DNA strand, while the lighter shades indicate expression on the negative DNA strand. The symbols represent the mean value for the tissue on either the positive (Δ) or the negative (○) strand.
(237 KB PDF)

Table S1 Sample Information (18 KB XLS)

Table S2 Numbers of Probes Classified as Expressed (19 KB XLS)

Table S3 Expression Divergence and Divergence-to-Diversity Ratios (29 KB XLS)

Table S4 Numbers of Probes Classified as Differently Expressed (19 KB XLS)

Table S5 Coordinates of Probes with Significant Expression Difference between Humans and Chimpanzees (Student's *t*-test *p* < 0.001, FDR < 0.05)
(357 KB XLS)

Table S6 Properties of Primers Used in Q-PCR Analysis (21 KB XLS)

### Accession Numbers

All primary expression data are publicly available at ArrayExpress (http://www.ebi.ac.uk/arrayexpress) under the accession number E-TABM-136.

## Abbreviations

FDR: false discovery rate
