# Supplementary material for: Functionality of Intergenic Transcription: An Evolutionary Comparison
Source: PLoS Genet. 2006 Oct 13;2(10):e171. doi: 10.1371/journal.pgen.0020171 (PMC1599769; doi:10.1371/journal.pgen.0020171)
Supplement: Supplementary file 1 [file pgen.0020171.sg001.pdf]

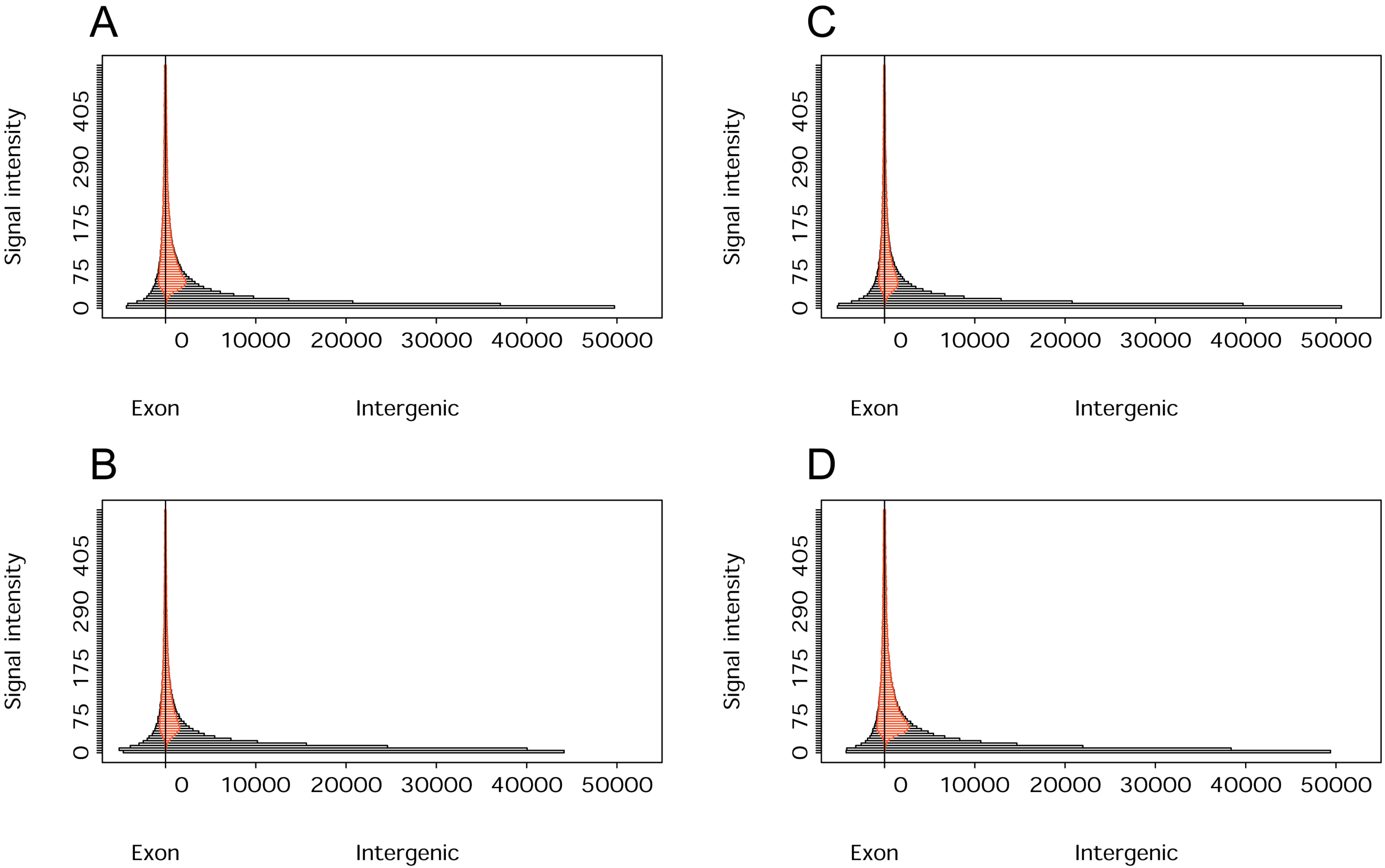
A
B
C
D
Signal intensity
0
75
175
290
405
0
10000
20000
30000
40000
50000
Exon
Intergenic
