# Supplementary material for: Functionality of Intergenic Transcription: An Evolutionary Comparison
Source: PLoS Genet. 2006 Oct 13;2(10):e171. doi: 10.1371/journal.pgen.0020171 (PMC1599769; doi:10.1371/journal.pgen.0020171)
Supplement: Supplementary file 2 [file pgen.0020171.sg002.pdf]

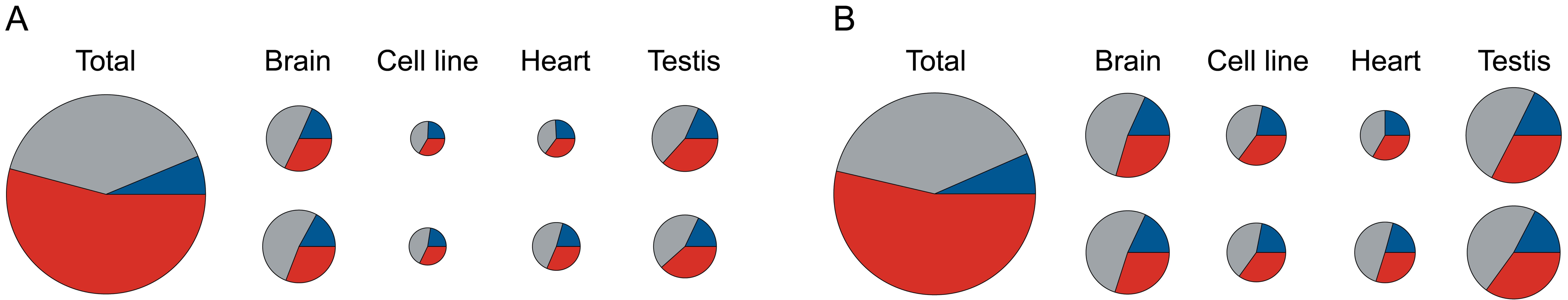
A
Total
Brain
Cell line
Heart
Testis
B
Total
Brain
Cell line
Heart
Testis
