# Supplementary material for: Functionality of Intergenic Transcription: An Evolutionary Comparison
Source: PLoS Genet. 2006 Oct 13;2(10):e171. doi: 10.1371/journal.pgen.0020171 (PMC1599769; doi:10.1371/journal.pgen.0020171)
Supplement: Supplementary file 3 [file pgen.0020171.sg003.pdf]

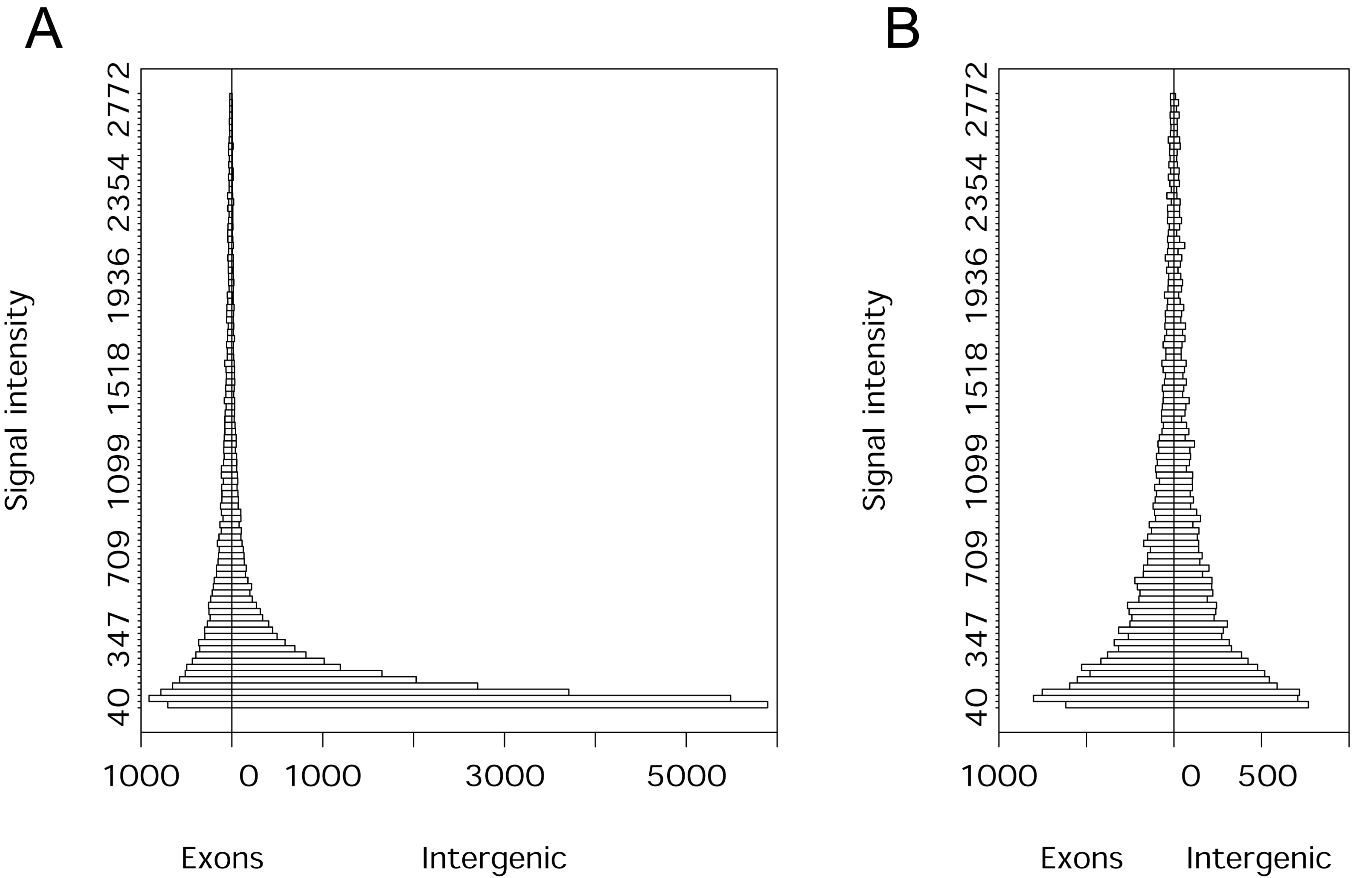

A
B
Signal intensity
40
347
709
1099
1518
1936
2354
2772
1000
0
1000
3000
5000
500
Exons
Intergenic
