# Supplementary material for: Functionality of Intergenic Transcription: An Evolutionary Comparison
Source: PLoS Genet. 2006 Oct 13;2(10):e171. doi: 10.1371/journal.pgen.0020171 (PMC1599769; doi:10.1371/journal.pgen.0020171)
Supplement: Supplementary file 4 [file pgen.0020171.sg004.pdf]

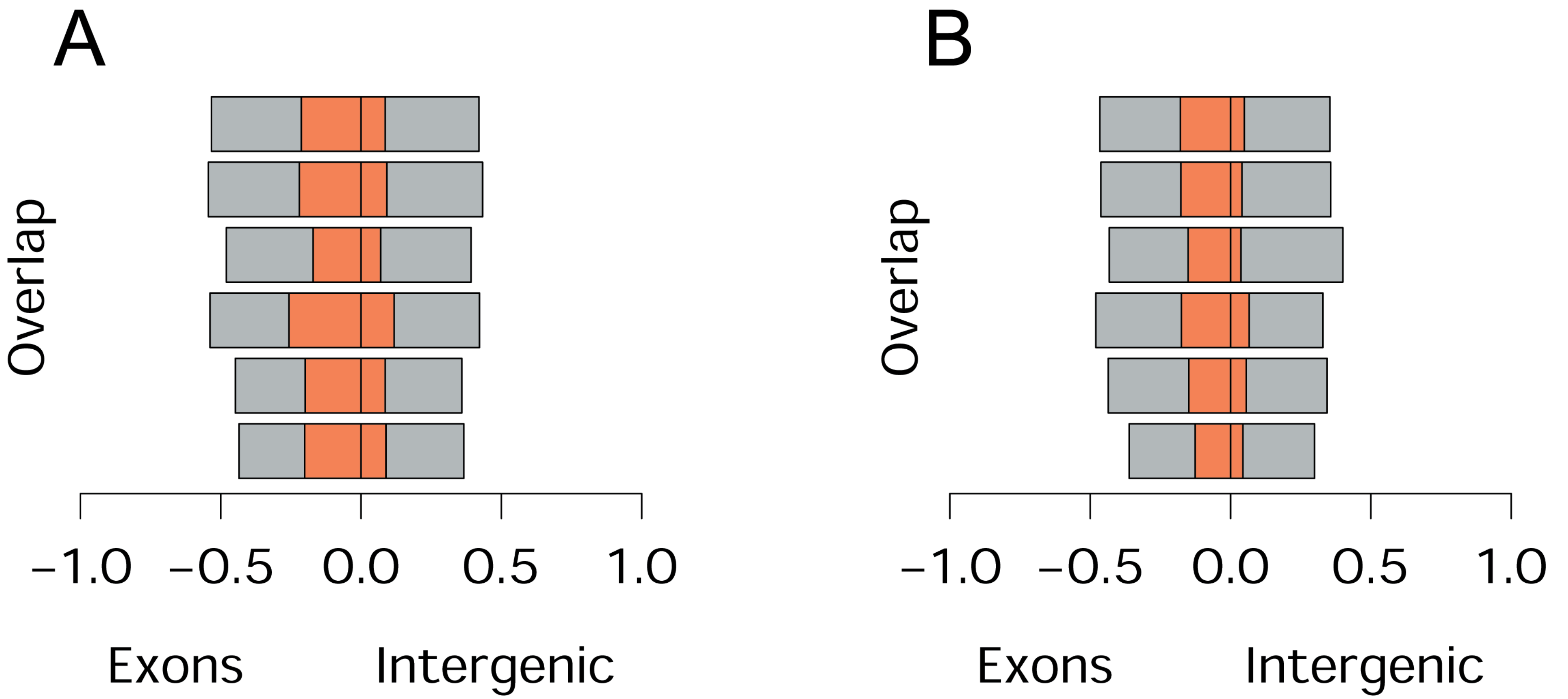
A
B
Overlap
Overlap
−1.0
−0.5
0.0
0.5
1.0
−1.0
−0.5
0.0
0.5
1.0
Exons
Intergenic
Exons
Intergenic
