# Supplementary material for: Functionality of Intergenic Transcription: An Evolutionary Comparison
Source: PLoS Genet. 2006 Oct 13;2(10):e171. doi: 10.1371/journal.pgen.0020171 (PMC1599769; doi:10.1371/journal.pgen.0020171)
Supplement: Supplementary file 5 [file pgen.0020171.sg005.pdf]

A

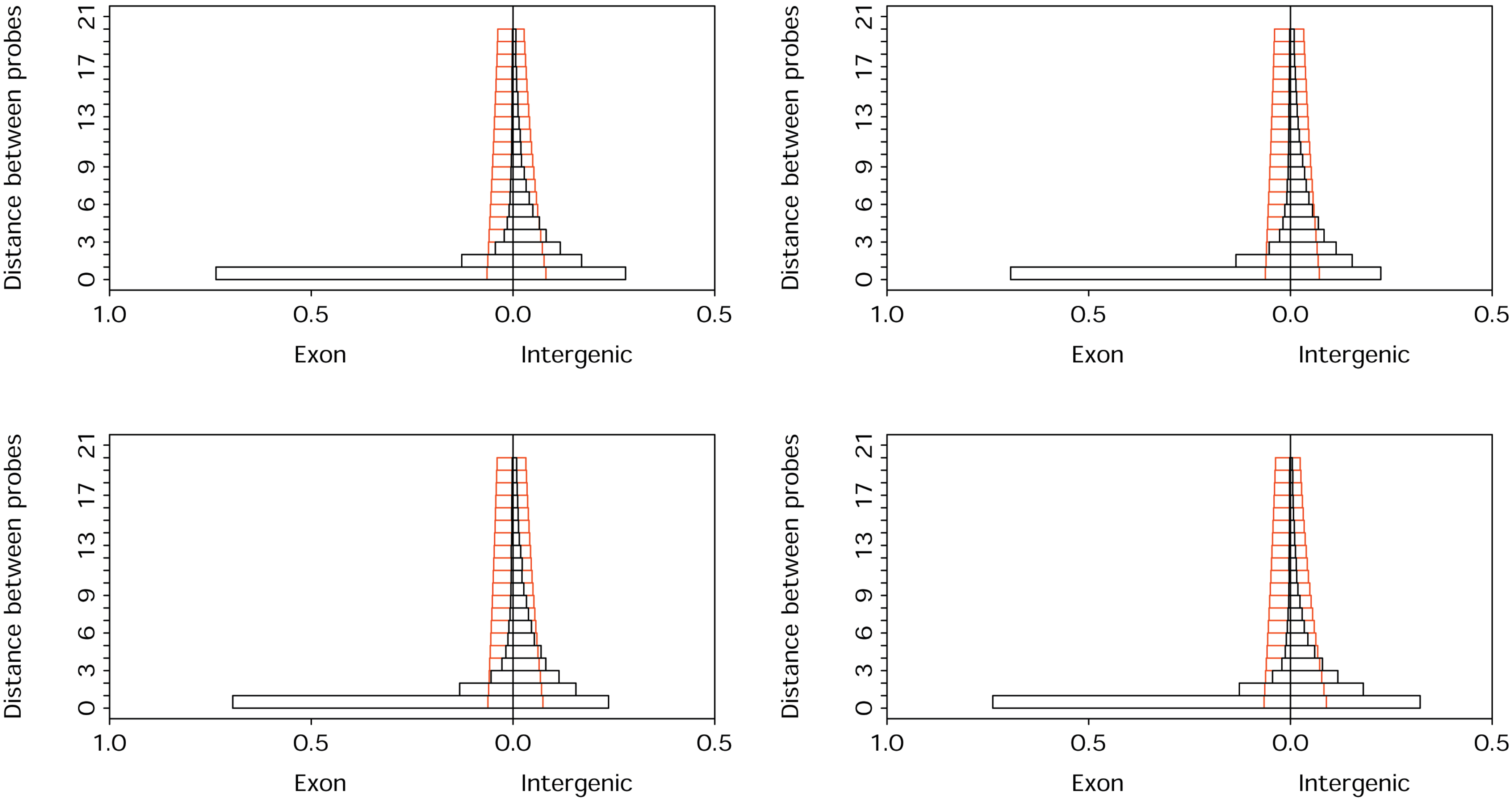
Distance between probes
0 3 6 9 13 17 21
1.0 0.5 0.0 0.5
Exon
Intergenic

B

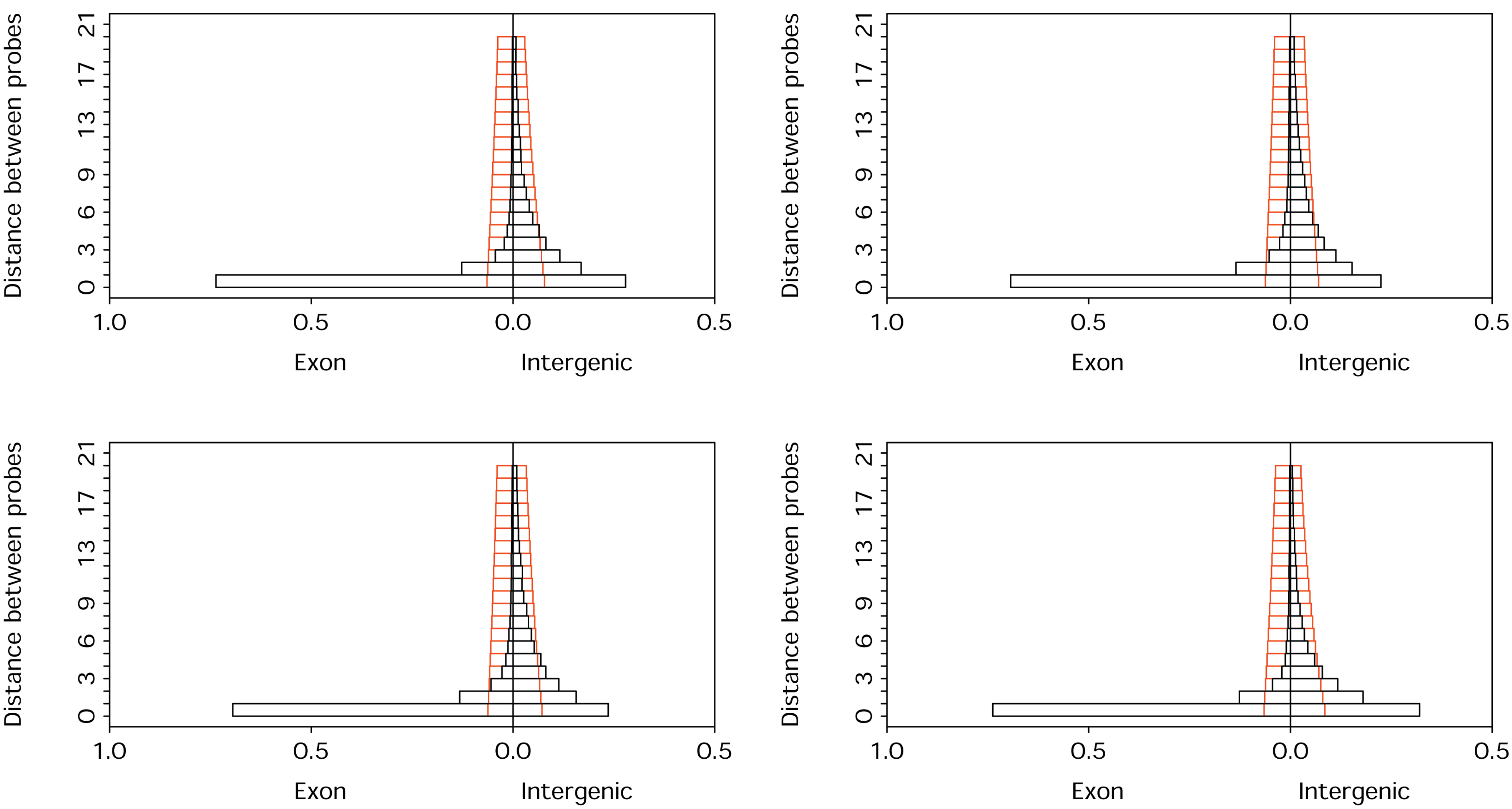
Distance between probes
0 3 6 9 13 17 21
1.0 0.5 0.0 0.5
Exon
Intergenic
