# Supplementary material for: Functionality of Intergenic Transcription: An Evolutionary Comparison
Source: PLoS Genet. 2006 Oct 13;2(10):e171. doi: 10.1371/journal.pgen.0020171 (PMC1599769; doi:10.1371/journal.pgen.0020171)
Supplement: Supplementary file 7 [file pgen.0020171.sg007.pdf]

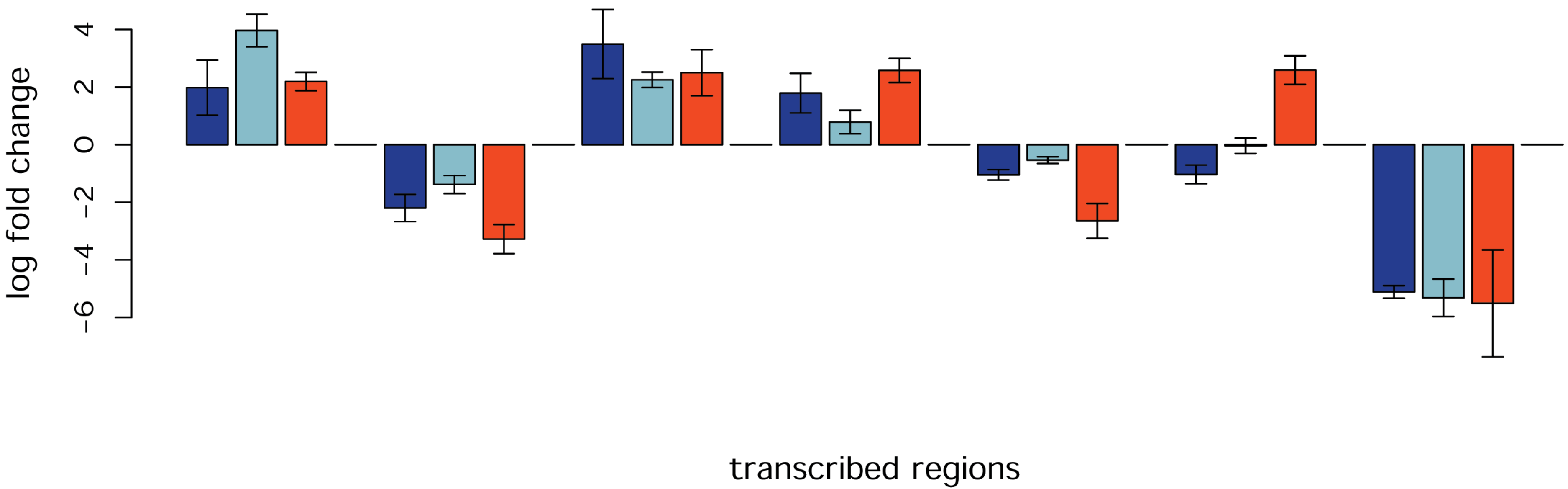

log fold change
4
2
0
−2
−4
−6
transcribed regions
