# Supplementary material for: Functionality of Intergenic Transcription: An Evolutionary Comparison
Source: PLoS Genet. 2006 Oct 13;2(10):e171. doi: 10.1371/journal.pgen.0020171 (PMC1599769; doi:10.1371/journal.pgen.0020171)
Supplement: Supplementary file 8 [file pgen.0020171.sg008.pdf]

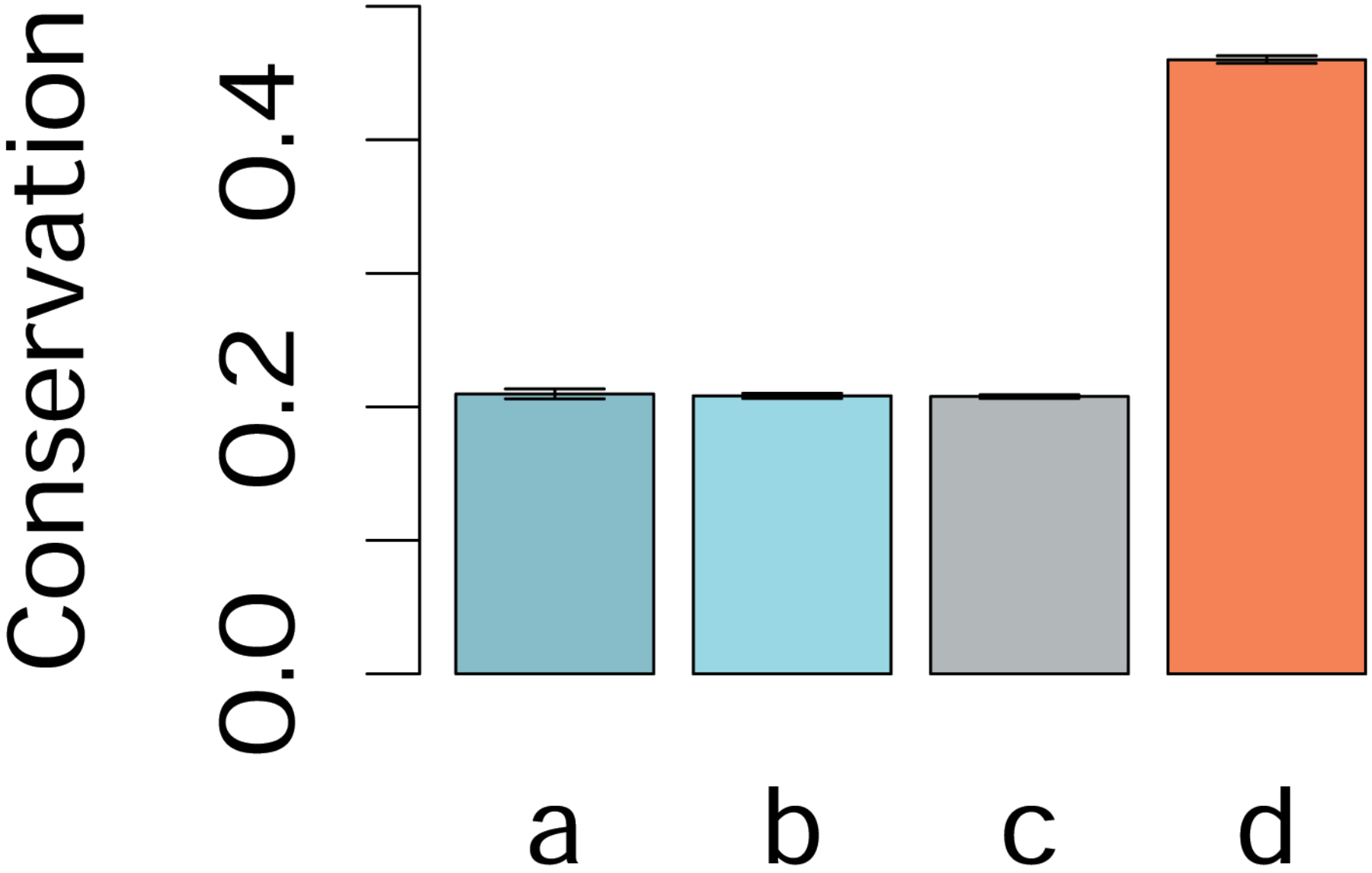
Conservation
0.0
0.2
0.4
a
b
c
d
