# Supplementary material for: Functionality of Intergenic Transcription: An Evolutionary Comparison
Source: PLoS Genet. 2006 Oct 13;2(10):e171. doi: 10.1371/journal.pgen.0020171 (PMC1599769; doi:10.1371/journal.pgen.0020171)
Supplement: Supplementary file 9 [file pgen.0020171.sg009.pdf]

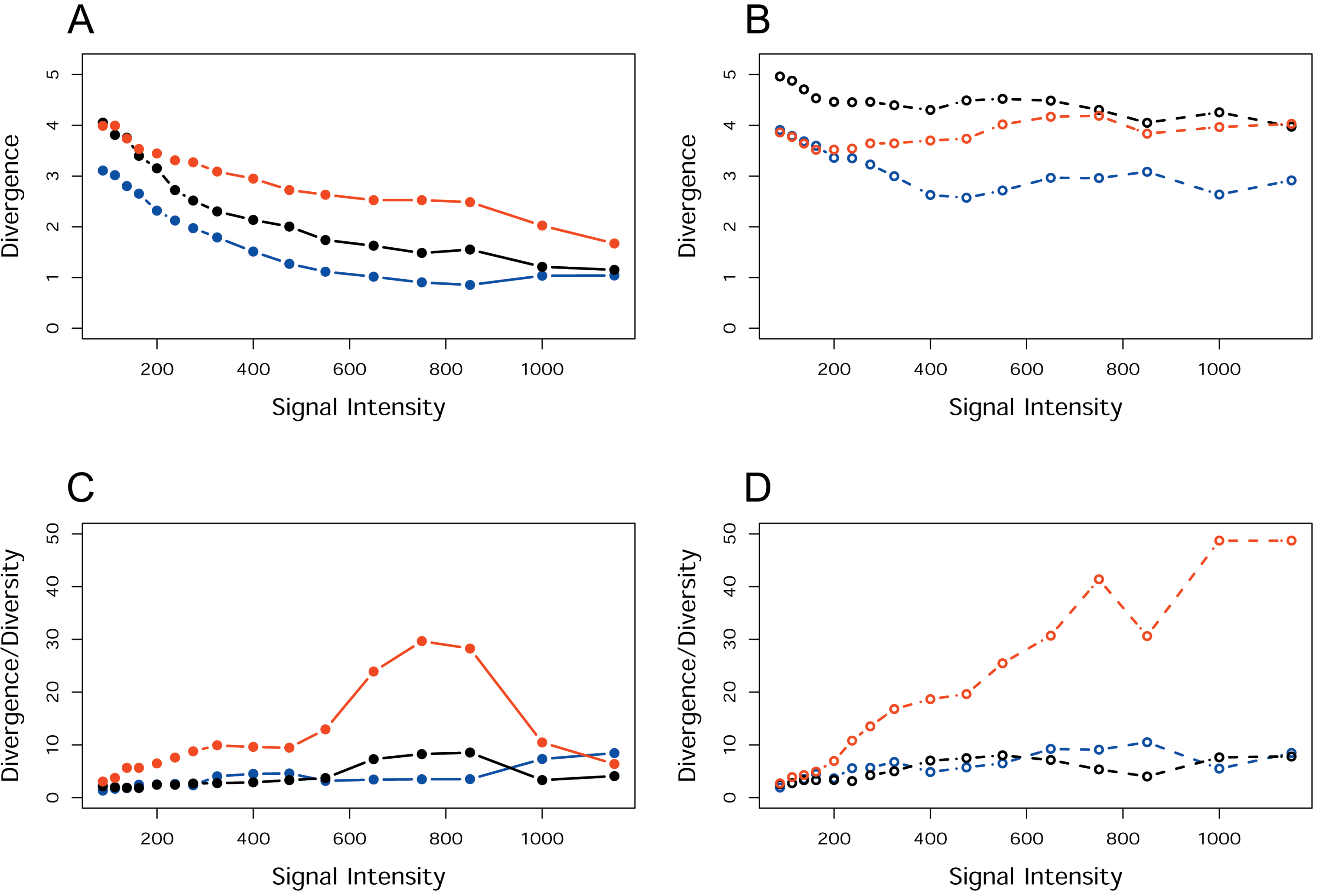
A
B
C
D
Divergence
Divergence/Diversity
Signal Intensity
0
1
2
3
4
5
10
20
30
40
50
200
400
600
800
1000
