# Supplementary material for: Functionality of Intergenic Transcription: An Evolutionary Comparison
Source: PLoS Genet. 2006 Oct 13;2(10):e171. doi: 10.1371/journal.pgen.0020171 (PMC1599769; doi:10.1371/journal.pgen.0020171)
Supplement: Supplementary file 10 [file pgen.0020171.sg010.pdf]

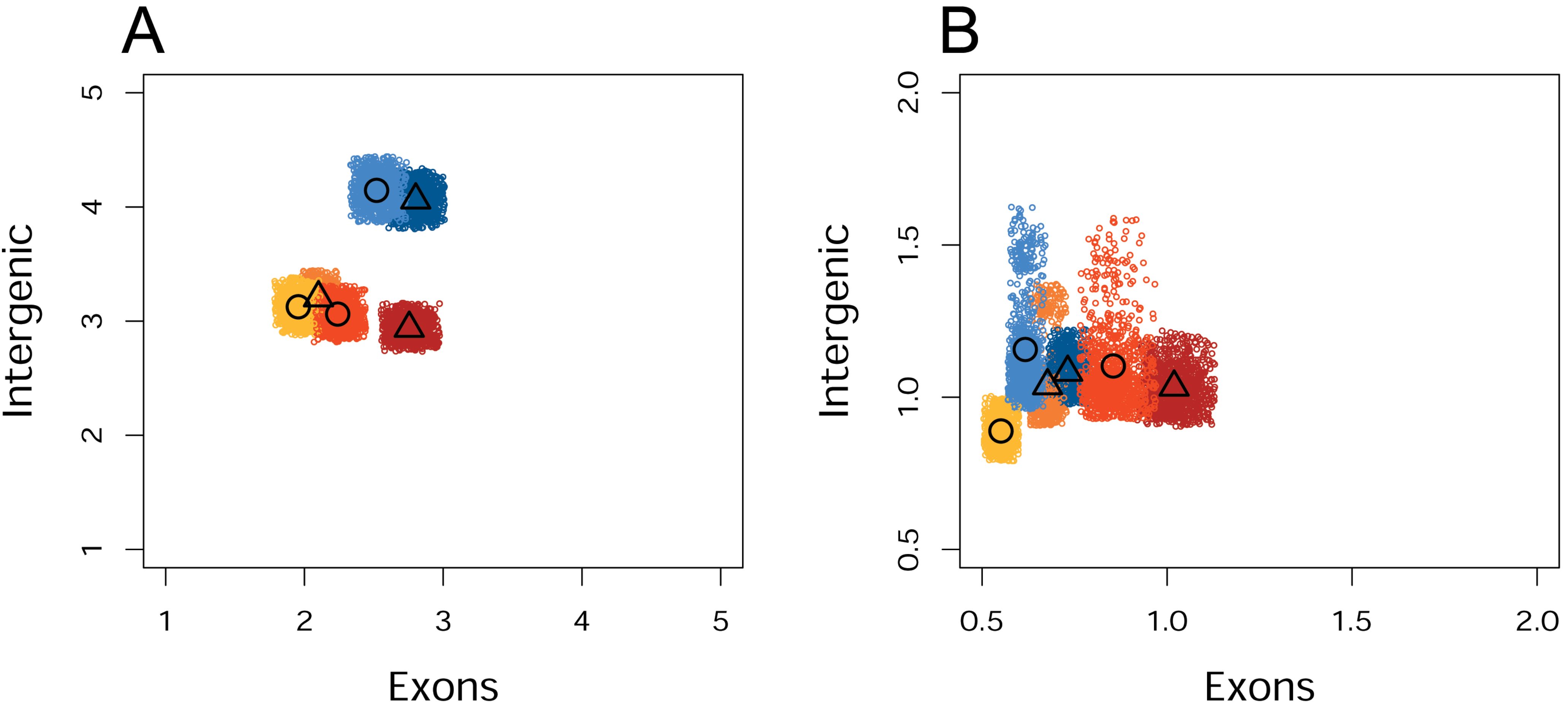
A
B
Intergenic
Exons
1
2
3
4
5
0.5
1.0
1.5
2.0
